# Measurement of the Surface Area of the Renal Sinus Fat Using MDCT: Correlation with Presence and Severity of Essential Hypertension and Body Mass Index

**DOI:** 10.5334/jbsr.2776

**Published:** 2022-10-07

**Authors:** Emad H. Abdeldayem, Mohamed G. Mansour, Basant M. Raief Mosaad

**Affiliations:** 1 Radiology Department, Faculty of Medicine, Ain Shams University, Cairo, EG

**Keywords:** Chronic kidney disease (CKD), cardiovascular disease (CVD), renal sinus fat area (RSFA)

## Abstract

**Objectives:**

Essential hypertension remains a major modifiable risk factor for cardiovascular disease. Excess visceral adipose tissue is associated with the presence of adverse metabolic risk factors. Our study aims to measure the surface area of the renal sinus fat using MDCT and correlate the renal sinus surface area with the presence and grading of essential hypertension as well as body mass index.

**Materials and Methods:**

This cross-sectional study included two groups; the patients’ group including 40 cases presented with a history of primary essential hypertension and the control group including 40 cases. The average of the surface area of the two kidneys as well as the average of the surface area of sinus fat was measured in the control and patient subgroups and was correlated with the presence and grading of essential hypertension as well as body mass index.

**Results:**

There was a significant correlation between the presence and grading of essential hypertension with prominent renal sinus fat. There was a significant correlation between the average surface area of kidneys and surface area of sinus fat in overweight and obese groups than in the control group (P < 0.01).

**Conclusion:**

Obesity is now recognized as a risk factor for the development of renal dysfunction. There was a significant correlation between the surface area of renal sinus fat measured using MDCT and the presence as well as grading of essential hypertension, suggesting that renal sinus fat may promote cardiovascular events.

## Introduction

Essential hypertension remains a major modifiable risk factor for cardiovascular disease (CVD) despite important advances in our understanding of its pathophysiology and the availability of effective treatment strategies [1].

Adipose tissue modifies the development of ‎cardiovascular disease in a complex manner [2,3], thus understanding the mechanisms by which visceral fat promotes cardiovascular events, would enable practitioners to target therapies to reduce cardiovascular events in individuals with high intra-peritoneal fat [4,5].

Renal sinus lipomatosis, which is a particular depot of visceral fat; involves prominent fat ‎proliferation that leads to mass effect on the intrarenal ‎collecting system. Computed Tomography and Magnetic ‎Resonance imaging directly reveal the fatty nature of ‎sinus lipomatosis [6,7,8].

The increased adipose tissue was associated with various metabolic abnormalities as well as the adverse cardiorenal outcome. For renal sinus fat (RSF), it was demonstrated that increased fat at this anatomical location is associated with hypertension as well as lower GFR, statistically independent of fat accumulation in other compartments [9].

Our study aims to measure the surface area of the renal sinus fat using MDCT and correlate the renal sinus surface area with the presence and grading of essential hypertension as well as body mass index.

## 2. Materials and Methods

### 2.1. Patients

This cross-sectional study included 40 patients from March 2019 to June 2021 who were recruited from the attendants of the cardiology clinic, at a multi-disciplinary private hospital. Participation in the study was voluntary after receiving informed consent. The study was approved by the institutional regulatory board of the Hospital. All procedures performed were under the ethical standards of the responsible committee on human experimentation (institutional and national) and with the Helsinki Declaration of 1975, as revised in 2008.

### 2.2. Inclusion criteria

- Recent diagnosis of primary hypertension, which was defined as the average of 2 or more diastolic BP measurements on at least 2 subsequent visits, was ≥90 mm Hg or when the average of multiple systolic BP readings on 2 or more subsequent visits was consistent ≥140 mm Hg [10].

### 2.3. Exclusion criteria

- Secondary causes of hypertension such as renovascular disease.
- Diabetes Mellitus.
- Other risk factor for hypertension as smoking.
- Family history of hypertension.

As the renal sinus surface area is variable with age, sex, race, body mass index, and visceral-adipose-tissue changes we were limited by the lack of standard normal reference value of renal sinus fat area (RSFA), as reported in Huang et al. [11], so a control ‎group of another 40 patients was selected.‎

The control group patients were normotensive and were selected from the same ‎medical institute. They underwent a computed tomography (CT) scan of the pelvi-abdomen for other diagnostic purposes to standardize the reference range. All the control group patients had a medical history free of any renal troubles and had normal size and CT appearance of both kidneys. The body mass index (height/weight) was within the normal range between 18.5 and 24.9.

### 2.4. Technique of non-contrast CT examination of the kidneys

- Patient position: supine position with arms elevated.
- CT machine: High speed 64 slice CT machine – Philips.
- Technique: Scan direction was craniocaudal in all patients. Scout was taken starting from the 10th thoracic rib or the shadow of the diaphragmatic copula down to the iliac crest.
- Patient preparation: no specific preparations were needed.
- CT parameters: we used 1.25 mm thickness, 0.625 mm interval, and 512 × 512 matrix size with a 1.75:1 pitch number. The rotation time used was 0.5 seconds with a tube speed of 35 mm/rotation. The Kv was 120 while the mA was ranging from 120 to 400 according to the body weight.

### 2.5. Post-processing measurements

**Figure 1:**
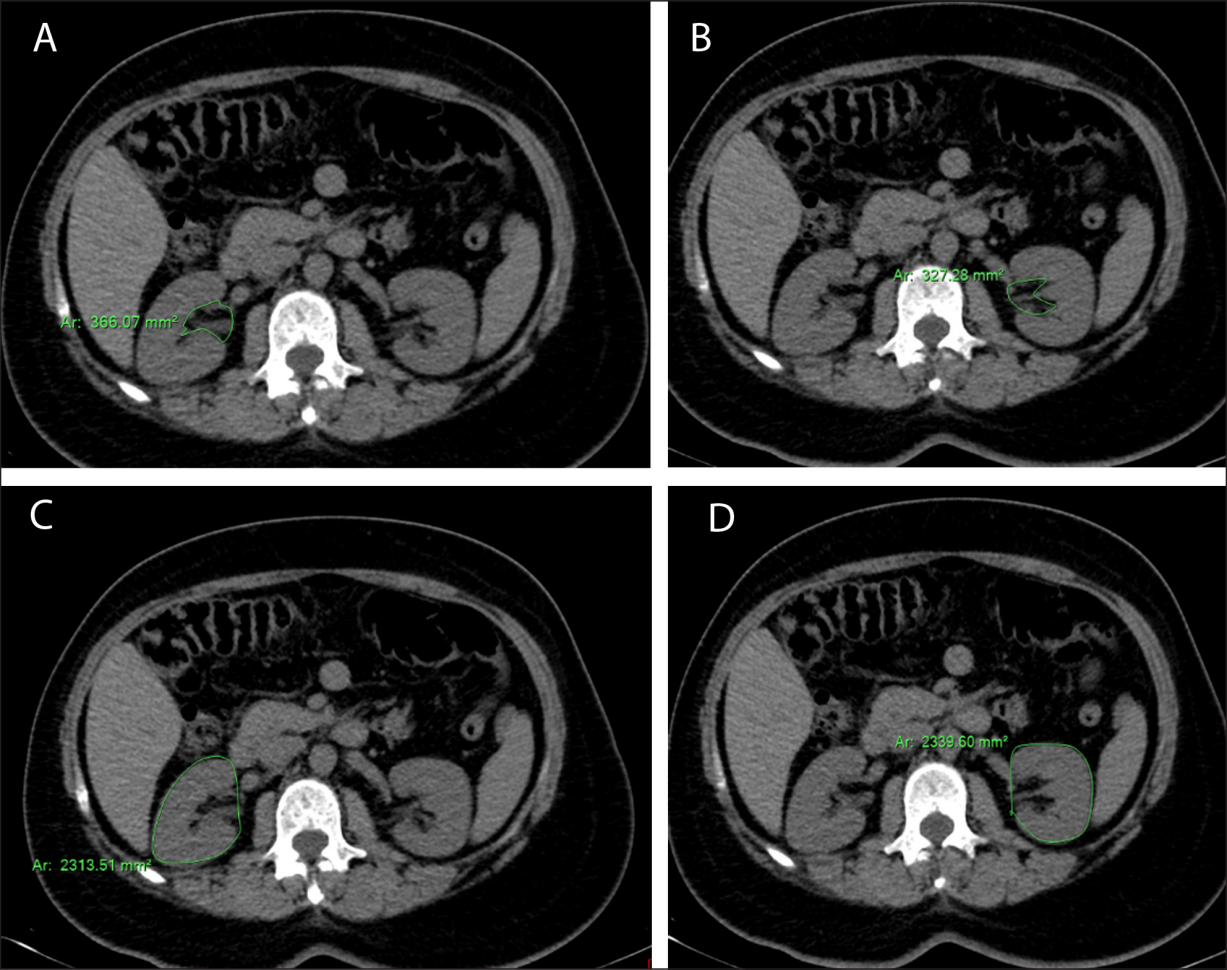
A 51-year-old male patient, with a recent history of essential hypertension. **A)** and **B)** The RSFA on the right side was 366.07 mm^2^ and on the left side was 327. 28 mm^2^. **C)** and **D)** The surface area of the right kidney and left kidney was 2313.51 and 2339.60 mm^2^ respectively.

**Figure 2:**
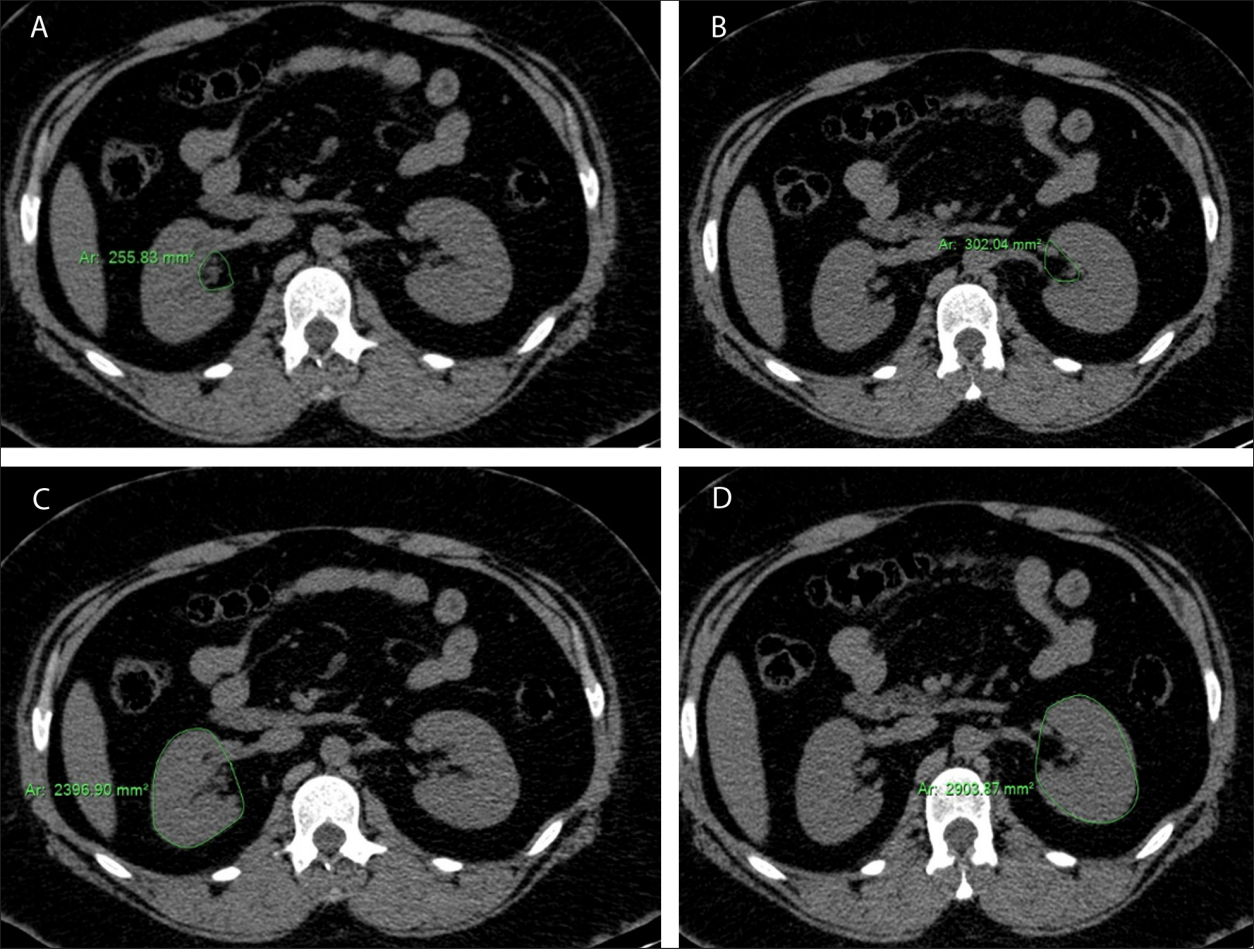
A control 57-year-old male patient, performed CT pelvi-abdominal examination with a history suggestive of appendicitis. **A)** and **B)** The RSFA on the right side was 255.83 mm^2^ and on the left side was 302.04 mm^2^. **C)** and **D)** The surface area of the right kidney and left kidney was 2396.9 and 2903.87 mm^2^ respectively.

- The slices at the mid-polar region of each ‎kidney containing the largest amount of sinus ‎fat based, on just visual inspection only, were determined.‎
- ‎We have selected the slice at the mid renal zone: ‎if the range included an odd number of slices, ‎then the middle slice of the range was traced. If the range included ‎an even number of slices, then the anatomically more ‎cranial of the two middle slices were traced.‎
- ‎Then, the sinus fat surface area was traced manually and identified by its low-density fatty ‎content with a negative Hounsfield unit (HU).‎
- Then, the surface area of each kidney was traced along the ‎renal tissue being demarcated by the surrounding retroperitoneal fat.
- The mean of the renal sinus fat surface area was calculated by summation of RSFA on both sides and divided by 2 to get the average of RSFA of every individual. The same steps were performed for the calculation of the mean of the surface area of the kidneys (Figures 1 and 2).

### 2.6. Data Management and Analysis

The collected data was revised, coded, tabulated, and ‎introduced to a PC using a statistical package for social sciences (IBM SPSS 20.0). Data were presented and suitable analysis was ‎done according to the type of data obtained for each parameter.‎

Descriptive Statistics

- ‎Mean, Standard deviation (+ SD), and range for ‎parametric numerical data, while Median and ‎Interquartile range (IQR) for non-parametric data.‎

Analytical Statistics

- ‎An Independent sample t-test was used to assess the ‎statistical significance of the difference of a parametric ‎variable between two independent means of two ‎study groups,
- ANOVA was used to assess the statistical significance of the difference of a parametric variable between means of more than two study groups.

## 3. Results

The patients’ group included 40 cases (42.2 ± 9.79 years of age), who presented with a history of primary essential hypertension and the second group was the control group including 40 patients (50.53 ± 13.65 years of age) (Table 1).

**Table 1:** Comparison between hypertensive patients and control group as regards demographic data.


VARIABLE		TYPE				CHI-SQUARE	P-VALUE

		CONTROLS		CASES			
	
		NO	%	NO	%		

**Sex**	**Male**	40	100.0%	40	100.0%		

**VARIABLES**		**TYPE**				**INDEPENDENT SAMPLE T-TEST**	**P-VALUE**

		**CONTROLS**		**CASES**			
	
		**MEAN**	**+ SD**	**MEAN**	**+ SD**		

**Age in years**		50.53	13.65	42.20	9.79	3.134	0.003**

**Height in cm**		175.38	6.61	171.05	8.07	2.623	0.010*

**Waist circumference**		91.73	5.84	100.47	10.89	-4.477	0.000**

**Weight**		72.15	6.58	80.20	15.70	-2.991	0.004**

**Body mass index**		23.40	.71	27.27	4.39	-5.503	0.000**


The average surface area of both kidneys in the patient and control groups was 3110.17 + 826.98 and 2499.72 + 478.86 mm respectively, while the average surface area of sinus fat was 392.50 + 374.25 and 265.30 + 114.77 mm respectively.

A comparative study was performed between the patient and control groups regarding the age, waist circumference, weight, and body mass index with demographic data in (Table 2).

**Table 2:** Comparison between cases and controls as regards the average surface areas of both kidneys and RSFA.


VARIABLE		TYPE				CHI-SQUARE	P-VALUE

		CONTROLS		CASES			
	
		NO	%	NO	%		

**Average of the surface area of both kidneys**		2499.72	478.86	3110.17	826.98	–4.040	0.000**

**VARIABLES**		**MEDIAN**	**IQR**	**MEDIAN**	**IQR**	**MANN WHITNEY U TEST**	**P-VALUE**

**Average of RSFA in mm^2^ of both kidneys**		265.30	114.77	392.50	374.25	426.000	0.000**

**The ratio between the average RSFA and average surface area of both kidneys.**		10.10	3.63	13.00	13.00	613.000	0.072


* Significant correlation.

The average surface area of both kidney as well as sinus fat was compared between the patient and control groups and found that both were significantly higher in the patient group than in the control group. The average of the surface area of the two kidneys as well as the average of the surface area of sinus fat was measured in the control and patient subgroups according to blood pressure (grade I, II, and III) and there was a significant correlation between the presence and grading of hypertension with prominent renal sinus fat (Table 3).

**Table 3:** Comparison of the average RSFA and average surface area of both kidneys between controls and patient subgroups according to blood pressure (grade I, II, and III).


VARIABLES	TYPE								ANOVA	P-VALUE

	CONTROLS		GRADE I HYPERTENSION		GRADE II HYPERTENSION		GRADE III HYPERTENSION			
			
	MEAN	+ SD	MEAN	+ SD	MEAN	+ SD	MEAN	+ SD		

**Average of the surface area of both kidneys**	2499.72	478.86	2774.98	483.12	2956.13	837.09	3538.56	893.01	9.354	0.000**

**VARIABLES**	**MEDIAN**	**IQR**	**MEDIAN**	**IQR**	**MEDIAN**	**IQR**	**MEDIAN**	**IQR**	**KRUSKAL WALLIS TEST**	**P-VALUE**

**Average of the surface area of sinus fat in mm^2^ of both kidneys**	265.30	114.77	272.00	395.00	415.00	360.00	544.20	316.00	17.958	0.000**

**The ratio between the average RSFA and the average surface area of both kidneys.**	10.10	3.63	9.00	10.00	14.00	13.00	13.00	10.75	6.201	0.102


* Significant correlation.

There was a significant correlation between the average surface area of kidneys and surface area of sinus fat in overweight and obese groups than in the control group (P < 0.01), suggesting obesity is more likely to be correlated with renal sinus lipomatosis, and thus increase the risk of essential hypertension (Table 4).

**Table 4:** Comparison of the average RSFA and average surface area of both kidneys between controls and overweight groups.


VARIABLES	TYPE								ANOVA	P-VALUE

	CONTROLS		NORMAL WEIGHT PATIENTS		OVERWEIGHT		OBESE			
			
	MEAN	+ SD	MEAN	+ SD	MEAN	+ SD	MEAN	+ SD		

**Average of the surface area of both kidneys**	2499.72	478.86	2471.38	469.72	3429.49	837.44	3405.07	766.20	13.981	0.000**

**VARIABLES**	**MEDIAN**	**IQR**	**MEDIAN**	**IQR**	**MEDIAN**	**IQR**	**MEDIAN**	**IQR**	**KRUSKAL WALLIS TEST**	**P-VALUE**

**Average of RSFA in mm^2^ of both kidneys**	265.30	114.77	368.00	375.50	440.00	553.75	415.00	337.50	13.648	0.003**

**The ratio between the average RSFA and average surface area of both kidneys.**	10.10	3.63	14.00	15.50	12.50	13.25	11.00	11.50	3.772	0.287


* Significant correlation.

## 4. Discussion

We have selected the surface area as a simple and available post-processing tool for the calculation of the sinus fat area as well as the surface area of the kidneys. The control of our study was selected for the setting of the reference range of renal sinus fat surface area and was from almost the same range of age.

Foster et al. [12] studied the volumetric renal sinus fat within the right and left kidneys separately and correlated their results with the renal sinus fat area within a single MDCT scan slice in the right kidney. They found a high correlation between volumetric and single-slice renal sinus fat in the right with p < 0.0001.

In animal models, excessive accumulation of fat within the renal sinus displaces and compresses the low-pressure renal lymphatics and veins as well as the ureters [13,14].

Also, renal sinus fat may exert an influence on hypertension ‎and CV risk through one of several mechanisms. Sinus lipomatosis‎ may compress the blood and lymph vessels in ‎the renal sinus with a consequent increase in the intrarenal hydrostatic pressure. This observation has been ‎previously shown in animal studies in which obese rabbits ‎exhibited larger kidneys with larger fat deposits ‎within the renal sinus. This occurred due to renal lymphatic ‎compression, despite an absence of detectable fat ‎accumulation within the renal parenchyma [15,16].

Various hypotheses emerged to explain how additional risk factors for hypertension as DM would affect the amount of RS fat. One of these hypotheses was an ectopic deposition of lipids into nonadipose tissues, such as the kidney. This can lead to the accumulation of toxic metabolites, derived from the metabolism of fatty acids. These metabolites may lead to mitochondrial dysfunction, apoptosis, and eventually renal injury [17].

Many classifications have been proposed for the subdivision of body fat into different compartments; most commonly, abdominal fat depots are classified into intra-peritoneal or visceral (IP) and subcutaneous (SC) fat compartments. Unlike SC fat, IP fat is associated with adverse cardiovascular (CV) events, including myocardial infarction and higher incidences of obesity-related hypertension.

In this study, we thought to address the association between renal sinus (RS) fat and hypertension for the following reasons: (1) those with higher amounts of IP fat also exhibit more body mass index; (2) increased amounts of RS fat have been associated with larger kidney size, (3) prominent RS fat is associated with the presence and grading of hypertension. Further studies are warranted to determine whether increased RS fat causes or contributes to poor control of hypertension.

## 5. Conclusion

Excess visceral adipose tissue is associated with the presence of adverse metabolic risk factors and cardiovascular disease. There was a significant correlation between the surface area of renal sinus fat measured using MDCT and the presence as well as grading of essential hypertension. Thus, RSF is an imaging feature that may suggest likelihood of essential hypertension.

## 6. Study Limitations

Segmentation of the renal sinus fat area can be possible based on artificial intelligence. Together with volumetric calculation, an additional quantitative value to the radiology report can be performed, thus it may be used as a ‘screening tool’ for essential hypertension.

## Data Accessibility Statements

All the datasets used and analyzed in this study are available with the corresponding author on reasonable request.

## Abbreviations

**RS:** renal sinus.

**IP:** intraperitoneal.

**SC:** Subcutaneous fat.

**RSFA:** renal sinus fat area.

**CKD:** Chronic kidney disease.

**CVD:** cardiovascular disease.

**CV:** cardiovascular.

**GFR:** glomerular filtration rate.

## References

[1] Carretero OA, Oparil S. Circulation. 2000 Jan; 101(3): 329–335. DOI: 10.1161/01.CIR.101.3.329 10645931

[2] Foster MC, Hwang SJ, Porter SA, et al. Fatty kidney, hypertension, and chronic kidney disease; the Framingham Heart Study. Hypertension. 2011; 58: 784–790. DOI: 10.1161/HYPERTENSIONAHA.111.175315 21931075 PMC3204377

[3] Karastergiou K, Fried S. Multiple adipose depots increase cardiovascular risk via local and systemic effects. Curr Atherosclerr Rep. 2013; 15: 1–11. DOI: 10.1007/s11883-013-0361-5 PMC3997174 23982264

[4] De Vries AP, Ruggenenti P, Ruan XZ, et al. Fatty kidney: the emerging role of ectopic lipid in obesity-related renal disease. Lancet Diabetes Endocrinol. 2014; 2: 417–426. DOI: 10.1016/S2213-8587(14)70065-8 24795255

[5] Foster MC, Hwang SJ, Larson MG, et al. Overweight, obesity, and the development of stage 3 CKD: the Framingham Heart Study. Am J Kidney Dis. 2008; 52: 39–48. DOI: 10.1053/j.ajkd.2008.03.003 18440684 PMC2531220

[6] Rha SE, Byun JY, Jung SE, et al. The Renal Sinus: pathologic spectrum and multi-modality imaging approach. Radiographics. 2004; 24: 117–131. DOI: 10.1148/rg.24si045503 15486236

[7] Abel N, Contino K, Jain N, et al. Eighth Joint National Committee (JNC-8) Guidelines and the outpatient management of hypertension in the African American population. N Am J Med Sci. 2015; 7(10):438–45. DOI: 10.4103/1947-2714.168669 26713289 PMC4677468

[8] Suwelack B, Kobelt V, Erfmann M, et al. Long-term follow-up of ACE-inhibitor versus beta-blocker treatment and their effects on blood pressure and kidney function in renal transplant recipients. Transpl Int. 2003; 16: 313–320. DOI: 10.1111/j.1432-2277.2003.tb00306.x 12759722

[9] Taler SJ, Agarwal R, Bakris GL, et al. KDOQI US commentary on the 2012 KDIGO clinical practice guideline for management of blood pressure in CKD. Am J Kidney Dis. 2013; 62: 201–213. DOI: 10.1053/j.ajkd.2013.03.018 23684145 PMC3929429

[10] James P, Oparil S, Carter BL, et al. 2014 Evidence-Based Guideline for the Management of High Blood Pressure in Adults. Report From the Panel Members Appointed to the Eighth Joint National Committee (JNC 8). JAMA. 2014; 311(5): 507–520. DOI: 10.1001/jama.2013.284427 24352797

[11] Huang H, Chen S, Yu W, et al. The association between renal sinus fat area and the progression-free survival in Chinese non-metastatic clear-cell renal cell carcinoma patients. Oncotarget. 2017; 8(39): 65481–65491. DOI: 10.18632/oncotarget.19012 PMC5630347 29029447

[12] Foster MC, Hwang SJ, Porter SA, et al. Development and reproducibility of a computed tomography-based measurement of renal sinus fat. BMC Nephrol. 2011; 12(52). DOI: 10.1186/1471-2369-12-52 PMC3198884 21970591

[13] Chughtai HL, Morgan TM, Rocco M, et al. Renal sinus fat and poor blood pressure control in middle-aged and elderly individuals at risk for cardiovascular events. Hypertension. 2010; 56(5): 901–906. DOI: 10.1161/HYPERTENSIONAHA.110.157370 20837881 PMC3634339

[14] Cross NB, Webster AC, Masson P, et al. Antihypertensives for kidney transplant recipients: systematic review and meta-analysis of randomized controlled trials. Transplantation. 2009; 88(1): 7–18. DOI: 10.1097/TP.0b013e3181a9e960 19584673

[15] De Pergola G, Campobasso N, Nardecchia A, et al. Para and perirenal ultrasonographic fat thickness is associated with 24-hour mean diastolic blood pressure levels in overweight and obese subjects. BMC Cardiovasc Disord. 2015; 15: 108. DOI: 10.1186/s12872-015-0101-6 26419359 PMC4588871

[16] Jordan J, Yumuk V, Schlaich M, et al. Joint statement of the European Association for the Study of obesity and the European Society of Hypertension: Obesity and difficult to treat arterial hypertension. J Hypertens. 2012; 30(6): 1047–1055. DOI: 10.1097/HJH.0b013e3283537347 22573071

[17] Tonelli M, Moyé L, Sacks FM, et al. Cholesterol and recurrent events trial investigators effect of pravastatin on loss of renal function in people with moderate chronic renal insufficiency and cardiovascular disease. J Am Soc Nephrol. 2003; 14(6): 1605–1613. DOI: 10.1097/01.ASN.0000068461.45784.2F 12761262

